# Using a nominal group technique to approach consensus on a resilience intervention for smoking cessation in a lower socioeconomic population

**DOI:** 10.1186/s12889-019-7939-y

**Published:** 2019-11-27

**Authors:** George Tsourtos, Kristen Foley, Paul Ward, Emma Miller, Carlene Wilson, Christopher Barton, Sharon Lawn

**Affiliations:** 1 Discipline of Public Health, College of Medicine and Public Health, Flinders University, Adelaide, South Australia Australia; 2 Flinders Centre for Innovation in Cancer, College of Medicine and Public Health, Bedford Park, Adelaide, South Australia Australia; 3 Olivia Newton John Cancer Wellness and Research Centre, Heidelberg, Melbourne, Victoria 3084 Australia; 4 Department of Psychology and Counselling, School of Psychology and Public Health, College of Science, Health and Engineering, Latrobe University, Melbourne, Victoria Australia; 5 Department of General Practice, Monash University, Melbourne, Victoria Australia; 6 Flinders Human Behaviour and Health Research Unit, College of Medicine and Public Health, Bedford Park, Adelaide, South Australia Australia

**Keywords:** Consensus, Resilience intervention, Smoking cessation, Lower SES populations, Nominal group technique

## Abstract

**Background:**

Smoking prevalence remains inequitably high for lower SES (socioeconomic status) populations. The psychosocial interactive model of resilience theorises that resilience might be ‘switched on’ in order to support and/or maintain smoking cessation for these populations. This study aimed to develop a Resilience Intervention for Smoking Cessation (RISC) through reviewing the extant literature around efficacious interventions for smoking cessation. Deliberative democracy principles were then used to understand lay perspectives regarding this potential smoking cessation program.

**Methods:**

Public health databases were searched to find efficacious psycho-social resilience interventions in the peer-reviewed literature for smoking cessation amongst lower SES populations. Potential components for RISC were selected based on evidence within the literature for their effectiveness. We then employed the Nominal Group Technique (NGT) to create discussion and consensus on the most socially appropriate and feasible components from the perspective of smokers from low SES areas. The NGT included 16 people from a lower SES population in southern metropolitan Adelaide who indicated they were seriously contemplating quitting smoking or had recently quit. Data were collected from multiple Likert ratings and rankings of the interventions during the NGT workshop and analysed descriptively. The Wilcoxon signed-ranked test was used where appropriate. Qualitative data were collected from participant reflections and group discussion, and analysed thematically.

**Results:**

Six smoking cessation interventions, likely to enhance resilience, were selected as potential constituents for RISC: mindfulness training; setting realistic goals; support groups; smoke free environments; mobile phone apps; and motivational interviewing. Consensus indicated that mindfulness training and setting realistic goals were the most acceptable resilience enhancing interventions, based on perceived usefulness and feasibility.

**Conclusions:**

This research applied principles from deliberative democracy in order to illuminate lay knowledge regarding an appropriate and acceptable smoking cessation resilience program for a lower SES population. This process of collaborative and complex knowledge-generation is critically important to confront inequities as an ongoing challenge in public health, such as smoking cessation for disadvantaged groups. Further research should involve development and trial of this resilience program.

## Background

Smoking prevalence is inequitably high for people living in lower socioeconomic status populations and who have low incomes in many countries including Australia. In 2014–15, 20% of people living in the most disadvantaged areas of Australia smoked compared to 7% in the least disadvantaged areas [1]. Brown et al.’s systematic review of smoking cessation interventions concluded that quit programs that have not specifically targeted lower socioeconomic status (SES) groups are more likely, overall, to lead to increased inequalities in smoking [2]. There is, however, promising evidence that the relative disadvantage of lower SES populations (in our study this means people with low incomes and living in lower socioeconomic areas, where multiple barriers to smoking cessation are likely to be at play at both the individual and area levels) can be mitigated if smoking cessation interventions are tailored to the specific requirements of this population [3]. In order to successfully develop and implement such programs, the voices from this target population need to be heard, understood, and allowed to meaningfully influence program design. This study aimed to engage with people from a lower SES population, hence we recruited smokers who reside in lower socioeconomic areas and have low incomes. We then asked them to discuss and reflect on what interventions they feel would work as part of a ‘resilience intervention’ for smoking cessation.

Relevant literature has identified resilience theory as an approach to address higher smoking rates among lower SES populations. The appeal of this approach is that it employs an asset-based focus rather than deficits [4–9]. The ‘resilience construct’ considers how an individual can draw upon resources when confronted with hardship, difficulties, and danger, in order to overcome negative life experiences [10] [11]. Previous studies have helped demonstrate that perceived stress and adversity are large barriers to smoking cessation for those who reside in lower SES populations [12–14]. Resilience is defined as ‘bouncing back from adversity’, as well as finding hope and meaning [15]. However, resilience studies have often achieved only modest smoking abstinence outcomes, and have mainly only focussed on youths (children, adolescents, and young adults) [8, 16–19].

Ward et al.’s psychosocial interactive model of resilience advances previous resilience work because it considers the external social environment in addition to the internal psychological properties of an individual, proposing that interaction between these influences resilience over the individual’s life-course [7]. The model has been used as a theoretical framework to explain how changes in the social environment (external resilience) can help activate or ‘switch on’ internal resilience properties (e.g. self-esteem, self-confidence, motivation, self-efficacy) by increasing social support to augment an individual’s ability to change behaviour. The model further explains how intervention strategies based on internal resilience (e.g. motivational interviewing) can be used to increase motivation and enhance self-belief. This is important for lower SES populations because these populations are associated with low self-efficacy, poor self-esteem, and low motivation to act, often due to lower social support and higher perceived stress [20–24]. Ward et al.’s model was introduced in relation to smoking and vulnerable groups, including those people who reside in postcodes with a lower socioeconomic status [4, 5, 7].

Although some studies in this area discuss resilience as protective [8, 16, 18, 19], none use the psychosocial interactive model as the foundation from which to develop interventions. Further, many studies in this field attempt to stop young people commence smoking [8, 18, 19], rather than working with adults that may have been smoking for an extended period of time, to quit. We therefore aimed to develop and tailor a ‘Resilience Intervention for Smoking Cessation’ (RISC) which would optimise the interaction between the internal psychological domains of an individual (e.g. self-esteem and confidence) and their external social environment (e.g. family ties) with a view to promote smoking cessation in adults from lower SES populations. We planned to identify validated interventions that improve internal resilience and utilise factors that promote a supportive environment (external resilience). This information will be used as the starting point for a conversation with representatives of the intended target population. This conversation was to focus on experience with, and acceptability of, these approaches to supporting smoking cessation and would utilise the Nominal Group Technique (NGT) to ascertain possible consensus.

## Methods

### Nominal group technique

We chose a NGT to hear the voices of smokers recruited from a lower SES population regarding the potential components of a resilience focused intervention program. The NGT facilitates and encourages the participation of all group members [25]. We used the NGT as a rigorous method to approach consensus on the usefulness and feasibility of potential constituent interventions for RISC, which we gained from the literature [25–27]. Below we describe how we compiled the list of potential components for the RISC, and then provide more detail about the way the NGT was applied in this research setting.

### Compiling a list of component interventions

**Fig. 1:**
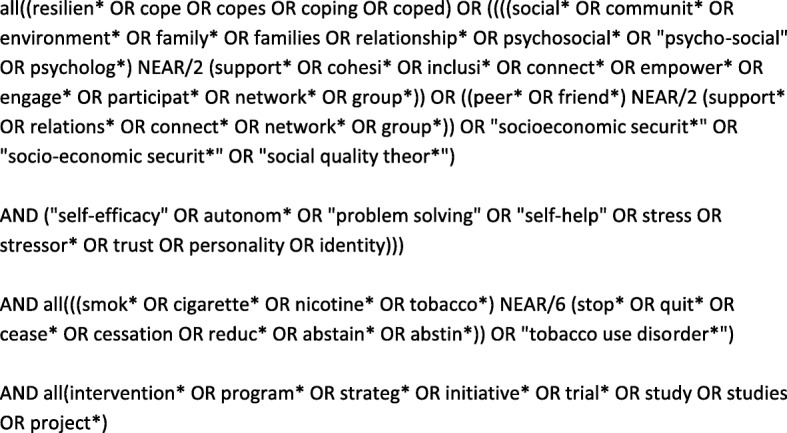
Search Strategy for PsycINFO, accessed via OVID (the syntax was adapted across 5 other databases)

In order to develop a list of interventions for discussion and review at our NGT workshop, a systematic search of the literature was undertaken to identify which interventions focussed on factors likely to optimise internal (psychologically based) and external (social environment) resilience and demonstrated an impact on smoking cessation. We developed a string of search terms that could capture studies exploring internal and external resilience for smoking cessation among adults from lower SES groups (an example search strategy is shown below in Fig. 1). It is important to note that cope and coping were utilised as potential synonyms for resilience on the basis that capacity to cope with challenges is a defining characteristic of resilience [4, 28].

**Fig. 2:**
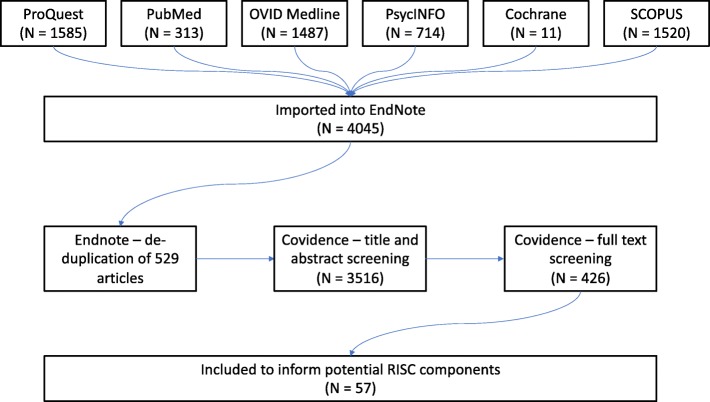
Process of systematic searching and review

The search syntax was adapted for 6 different databases. Databases searched and results retrieved included; ProQuest (1585), PubMed (313), Cochrane [11], OVIDMedline (1487), OVID PsycINFO (714) and SCOPUS (1520). In total, 4045 articles were imported to EndNote before de-duplication occurred, leaving 3516 articles (i.e. 529 duplicates removed). These articles were imported into Covidence, an online resource management tool which forms a component of the Cochrane review process (https://www.covidence.org/home). One researcher (KF) reviewed the title and abstract for relevance, subsequently including/excluding them from the study. This process removed 3033 articles, with 426 articles proceeded to full-text screening. Two researchers then reviewed the full-text of these 426 for relevance (KF and GT). Articles were excluded if the target population included primarily children or adolescents (less than 18 years of age), the written language was other than English, or the main study aim was to prevent smoking uptake. This process is shown diagrammatically in Fig. 2, below.

From the 57 articles included, potential interventions for inclusion in RISC were compiled. Six psycho-social interventions across the 57 articles were found to have evidence in support for their use and were subsequently earmarked for inclusion in our potential RISC and NGT discussion: Mindfulness Training; Motivational Interviewing; Setting Realistic Goals; Smoke-Free Environments; Mobile Phone Apps; and Support Groups. These interventions are outlined below in Table 1. The evidence from the academic literature was critically reviewed and analysed by the authors.

**Table 1:** Intervention description, purpose, evidence, and factors influencing use

Strategy	Description	Purpose	Evidence	Influencing Use
Mindfulness Training (MT)	Can be developed through meditation and designed to become self-aware of own reactions. MT could be delivered via professional counselling (e.g. psychologist) at subsidised cost and/or free online materials.	Cope with cigarette *cravings, urges, and stress (can all present as significant barriers to quit smoking).*	[29, 30]	Cost of professional support. However, there are free online materials.
Motivational Interviewing (MI)	A counselling approach used to help a smoker resolve uncertainty between wanting to smoke and quit. Quitline offers a free service.	To help smokers who are thinking of quitting increase and maintain their level of *motivation, confidence, and self-belie*f to quit.	[31–33].	Quitline is a professional support telephone service,which provides free motivational interviewing.
Setting Realistic Goals (SRG)	Developing a clear and specific plan of how quitting will happen, e.g. peer support for stressful periods. Could be delivered with the help of professional support, to help break-down steps.	Break down steps to quitting, makes goals more achievable, which builds *confidence*, *self-belief and motivation* to quit.	[23, 34–36]	Relatively inexpensive. Professional support can help with breaking down steps.
Smoke-free Environment (SFE)	Places or groups where smoking does not happen, e.g. sports clubs.	More healthful social networks and relationships that can help reinvent a *non-smoker identity.* Face fewer smoking triggers.	[7].	No financial costs. Decision may be made to leave behind friends, because they smoke.
Mobile Phone Apps	As an example, Text2Quit is a mobile phone text message app that includes social support, tracking progress, and giving tips on cravings [37].	Tailor messages for user and send timely messages. Tools for self-monitoring, and reminders. Helps cope with *cravings and can improve self-esteem, self-belief, confidence, and motivation to quit.*	[38, 39]	Access to Smartphone. Knowledge and ability to download mobile phone apps. Cost of app. Can connect to support groups.
Support Groups (SG)	Smokers contemplating quitting, meeting together. Participants offer emotional support and encouragement to quit. Trained ex-smoker can be included to act as a group facilitator/peer mentor.	Enhance *self-esteem* and lessen stress. Building self-belief, *confidence* and *motivation* to quit. Trained ex-smoker facilitator is a role model, that helps build *self-belief* in quitting.	[40, 41]	May require attending multiple support group sessions

To determine if any of these potential intervention components would be useful and feasible for individuals in our targeted population, a contextualised understanding of these interventions in relation to target population perspectives was critical. When designing a complex health intervention, Campbell et al. note that context is important and that the wider socioeconomic background should be considered [42]. Thus, smokers from lower SES populations must be meaningfully engaged in the development of interventions so they can be tailored to their needs. We describe here how we recruited individuals from these contexts to participate in our NGT and then describe the NGT in-depth.

### Sampling and recruitment

Purposive sampling was used to recruit adult female and male current smokers with low incomes and living in the lowest socioeconomic quartile in the southern metropolitan region of Adelaide, South Australia. Postcodes, which provide a broad indication of socioeconomic status (SES), were used to assign a Socio-Economic Index for Areas (SEIFA) [43] category to each participant. SEIFA is a measure used by the Australian Bureau of Statistics to rank communities in Australia based on their SES. We placed flyers and posters in shopping hubs in inner and outer-southern Adelaide metropolitan areas within the lowest socioeconomic quartiles as measured by SEIFA. We also advertised the study on Facebook to users who frequented these areas (‘location targeting’). The posters and flyers invited smokers or ex-smokers from low income households (less than $450 AUD income per person, per week) to contact the study research officer to express interest in participating in the study ($450 AUD is a similar amount to the calculated ‘poverty line’ described by the Australian Council of Social Service in 2018, the same year the data was collected for this study) [44]. Therefore, SEIFA was used as a measure to identify low SES metropolitan areas, whereas the posters and flyers more directly recruited individuals with low SES at the household level within those areas. When people telephoned to volunteer for the study, we described the NGT process to them and advised that we would send out an information pack for them to review. We also undertook snowball sampling, asking if they knew any other people who might like to attend.

To be eligible for the study, participants needed to have either smoked regularly for at least the past 2 years and self-identify as ‘seriously’ contemplating quitting or have quit within the last year. In total, we recruited 16 participants; 14 who were seriously thinking about quitting and 2 who had recently quit. An information pack that described the six resilience interventions was posted out to participants in the week prior to the NGT workshop. The information pack included some detail about the NGT and a half page of lay language information for each resilience intervention, which included providing evidence for each intervention (available on request from the corresponding author). Participants were encouraged to read through this information and write down any notes or questions they had to support discussion on the day of the workshop.

### Nominal group technique workshop

**Fig. 3:**
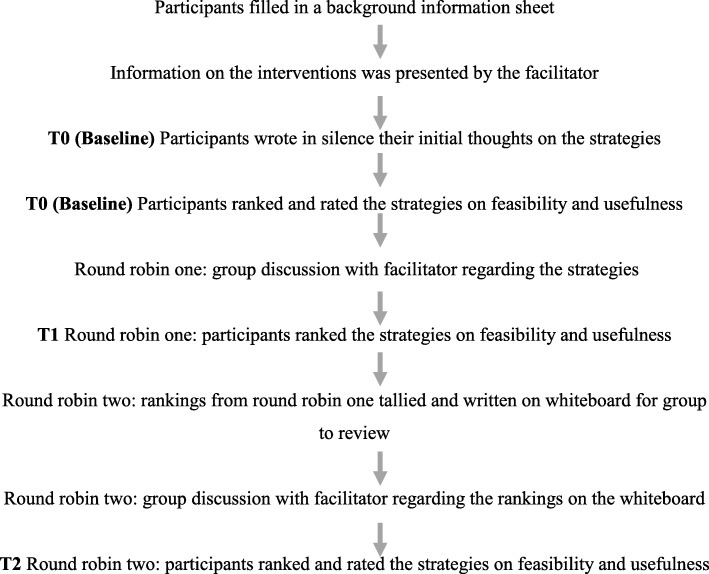
NGT Workshop procedure: step by step

The one-day NGT workshop was held in the southern suburbs of Adelaide at a place that could be easily accessed by public transport. Participants received $80 (AUD) to recompense any expenses associated with their attendance. In addition to the 16 participants, four members of the research team were present (including the facilitator). The NGT workshop was highly structured and the sequence of events is shown diagrammatically in Fig. 3. At commencement of the workshop participants completed a one-page background information sheet (developed by the authors, see Additional file 1: Figure S4) that included questions about their age, gender, smoking behaviour, and whether they had tried any of the six proposed interventions or any of the nicotine replacement therapies before (a list of these 7 options where presented and the respondent had to reply yes/no). The facilitator (SL) then verbally described the six resilience interventions briefly to the participants, reiterating general definitions and giving equal time and emphasis to each to avoid communicating favour towards any particular intervention. Both the information pack and the briefing delivered by the facilitator at the commencement of the NGT workshop made clear that participants were not invited to receive a yet to be developed and trialled RISC as part of this particular study.

Three rounds of intervention evaluation and two rounds of discussion were undertaken (see Fig. 3). Participants initially recorded baseline rankings (developed by the authors, see Additional file 2: Figure S5) of how useful each of the six interventions were (*most useful* (ranked 6) *to least useful* (ranked 1)), and rankings of how feasible to put into practice each of the six interventions were (*easiest* (ranked 6) *to most difficult* (ranked 1)). Participants rated at baseline (T0) for each of the six interventions, on a 9-point Likert scale (developed by the authors, see Additional file 3: Figure S6), the level of usefulness (9 represented the intervention would be *very useful* in helping them quit smoking and 1 represented *not at all useful*), and the level of feasibility (9 represented the intervention was *easy* to put into action or practice and 1 represented the intervention was *difficult to put into practice*). The participants were then asked to write down ‘in silence’ their thoughts on each of the six resilience interventions presented. These baseline rankings, Likert rankings and silent reflections were undertaken by participants before any group discussion, to understand initial ideas and views on the six interventions.

The first round robin of participant discussions followed; participants shared their thoughts on the six resilience enhancing interventions for approximately 90 min. The facilitator aimed to probe additional discussion from any quiet members in the group and ensure the group discussion stayed focused around evaluating the six interventions. The second rankings (T1) on usefulness and feasibility were completed at the end of this first round robin session and were then posted on whiteboards for group discussion in round robin two. The second and final round robin deliberations (75 min) were centred around the whiteboard postings of the rankings, with a focus on any large discrepancies between individual rankings, unexpected outcomes, or changes between rankings between T0 and T1. The NGT workshop was then concluded by collecting the final quantitative data (T2; third rankings and second Likert ratings).

The quantitative data were collected on paper. The qualitative data from group discussions were audio- and video-recorded to ensure it would be clear who was talking at which point. Both group discussions were fully transcribed for data analysis by one researcher from the team who was present on the day, to help with familiarity of participants voices. Which of the 6 interventions that might be accepted for RISC was discussed and explored between the facilitator and the participants at the end of the NGT workshop.

### Ethical approval

This research was approved by the Social and Behavioural Research Ethics Committee at Flinders University (project number 7747).

### Data analysis

All quantitative data were analysed using SPSS (IBM Corp. Released 2013. IBM SPSS Statistics for Windows, Version 22.0. Armonk, NY: IBM Corp.). This included descriptive analysis (means, medians, frequencies) of the participants’ background data as well as the Likert ratings and rankings of the interventions’ usefulness and feasibility [highest possible total ranking was 96 (16 participants x highest individual possible ranking of 6)]. The Wilcoxon’s signed-ranked test was used to establish if there was a statistically significant change in ratings of each intervention between baseline (T0) and post-deliberation (T2). Descriptive statistical analyses were employed to calculate where consensus might have been approached in terms of total ranked scores, percentage of high and low ranked scores, and Likert ratings.

Qualitative data were used to unpack detail for the ranking and rating decisions that participants made, and also understand the reasons behind the level of acceptability for each of the interventions. Further, these data helped understand and contextualise the reasons for consensus. All qualitative data were analysed for patterns and emerging themes (data-driven).

Coding of the round robin discussion transcriptions and writing ‘in silence’ data was undertaken independently by six members of the research team (three male and three female experienced researchers). These researchers then met twice to collaborate on how to interpret the data. Their different perspectives were triangulated to add rigour. Where the different analysts’ perspectives did not concur, they discussed and debated these differences until agreement was achieved. A constant comparison method was used to identify similarities and differences within and between the transcripts and the writing ‘in silence’ data [45].

## Results

### Quantitative data analysis: T0 (baseline), T1 (after first discussion) and T2 (end of workshop)

There were 14 current smokers who were seriously contemplating quitting and two ex-smokers, with a median 47.5 (*N* = 16) years of age across the total sample. Twelve participants were female. The median age of participants when they commenced smoking was 16.0 years of age (N = 16). The median number of cigarettes smoked per day for the current smokers was 10.5 (*N* = 14). The main reasons for wanting to quit smoking were related to health and financial expense of smoking. Twelve of the 14 current smokers confirmed that they had made at least one quit attempt, with two current smokers managing to reduce the amount they smoked but had not completed smoking cessation. See Table 2 regarding the full demographic and smoking history details for each participant.

**Table 2:** Individual participants’ demographic and smoking history details

Pseudo-nym	Sex	Age Range	Age when started smoking	Average number of cigarettes smoked per day	Current smoking status	Previous quit attempts	Reasons for quitting now
(Ariana)	F	40–50	17	20	Smoker	Nicotine Replacement Therapy (NRT) whole of June 2018.	Health and financial reasons.
(Lucy)	F	50–60	21	Normally 10, more if stressed (up to 20)	Smoker	Tried to quit 3 times. Birth of 2 children ~ 30 years ago (5 years and 8 years without smoking). Plus 5 years ago for a year.	Good health and financial.
(Paul)	M	50–60	13	Now 8–12	Smoker	Several times, up to 11 months, mostly by fitness training and also meditation, motivational books and affirmations.	Health, money, stench, don’t like being dependent.
(George)	M	50–60	16	6–10	Smoker	6 years ago for 2 years using neuro blocking tablets.	New found passion requires clear breathing underwater (scuba diving).
(Angela)	F	20–30	15	15	Smoker	On day 2 without smoking	–
(Veronica)	F	60–70	40	30	Smoker	–	Financial.
(Megan)	F	40–50	13	10	Smoker	–	To be healthy.
(Elizabeth)	F	40–50	16	15–20	Smoker	Yes on Champix, gave up for 3 months.	Health.
(Maria)	F	40–50	13	10 or more	Smoker	Yes at 40 years old for 2 years.	Turning 50.
(Jason)	M	30–40	16	7	Smoker	Reduce. Not quit.	Too expensive.
(Sophie)	F	30--40	15	2	Smoker	I was able to reduce the amount I smoked from approx. 10, to 4, to 2.	Start a family.
(Zoe)	F	60–70	19	15	Smoker	3 days.	Social outcast (‘treated less than human’). Cost is driving people to cheaper drugs – like ice/meth/grass.
(Andrea)	F	40–50	14	15	Non-smoker	With patches and support from family, I quit 5 months ago, still not smoking.	–
(Raymond)	M	30–40	13	15–25	Non-smoker	2010 with Champix for 6 months. April 2018, nicotine patches still not smoking.	–
(Evonne)	F	40–50	21	10–12	Smoker	NRT– 1.5 years.	Poor cardiovascular health.
(Sonia)	F	50–60	16	20	Smoker	Longest was 1 year.	Money, illness.

All participants had previously used smoking cessation interventions, which are shown below in Table 3. The most commonly used cessation interventions were; nicotine replacement therapy, setting realistic goals, and being engaged in smoke free environments. It was clear from the group discussions that past experience with cessation interventions influenced participant perspectives of all six interventions. For example, current smokers mostly perceived mobile phone apps in a negative light, based on previous experience.

**Table 3:** Cessation interventions previously employed by participants

	Motivational Interview	Support Group	Mindfulness Training	Realistic Goal Setting	Smoke Free Environ	Mobile Phone Apps	NRT
Smokers *N* = 14	1	5	5	9	8	4	10
Ex-smokers *N* = 2	0	0	0	2	2	2	2
Total	1/15	5/15	5/15	11/16	10/16	6/16	12/16

The Likert ratings measured usefulness and feasibility for each of the six interventions. These were measured at T0 and again at the end of the workshop (T2). They are shown in full in Table 4, and the findings summarised below.

**Table 4:** Likert rating median scores (1–9) for Usefulness and Feasibility at T0 and T2 (*N* = 16)

	USEFULNESS		FEASIBILITY	
	T0	T2	T0	T2
Mindfulness	7.0	9.0	4.0	7.0
Setting Realistic Goals	7.5	8.0	5.0	8.0
Mobile Phone Apps	2.5	3.0	4.0	6.0
Motivational Interviewing	5.5	4.0	3.0	5.0
Smoke Free Environments	7.5	7.0	3.5	6.5
Support Groups	6.0	5.0	2.0	3.0

### Findings from Likert ratings: usefulness

Apart from the mobile phone apps cessation interventions, which was rated relatively low, most interventions were reported as moderate to high (a score of 5 or higher) on usefulness at both T0 and T2 measures. The Likert median rating score for Mindfulness Training increased significantly from T0 to T2 [Wilcoxon Signed Rank Test, Z = -2.86, *p* < .01, *N* = 16]. Mindfulness Training was reported as being the most useful at T2, achieving the maximum score. Setting Realistic Goals was the second highest rated cessation intervention regarding usefulness at T2.

### Findings from Likert ratings: feasibility

Setting Realistic Goals was rated the most feasible intervention option at T2, with a large, although non-significant, increase in median score from T0. Mindfulness Training was the second highest rated for feasibility at T2. All interventions were rated, at T2, as moderate to high for feasibility, with the exception of Support Groups. The vast majority of participants rated Mindfulness Training (*N* = 16; 100%) and Setting Realistic Goals (*N* = 14, 87.5%) as highly useful (7 or more); and Mindfulness Training (*N* = 12; 73.2%) and Setting Realistic Goals (*N* = 13; 79.9%) as highly feasible (7 or more) at T2.

### Findings from rankings: feasibility and usefulness

Participants were also asked to rank the interventions on three occasions (T0, T1, and T2) in order from most feasible to least feasible and most useful to least useful. Table 5 summarises the total ranked scores for each of the six RISC interventions. Mindfulness Training and Setting Realistic Goals had the highest total rank scores at T2, for both feasibility (68 and 76) and usefulness (90 and 77). The usefulness total ranked score for Mindfulness Training increased considerably from T0 to T2, suggesting some participants changed their perspectives over the course of the day. There was also an increase in frequency count of participants who gave the highest ranking of six for usefulness to either Mindfulness Training or Setting Realistic Goals from T0 (*N* = 10) to T2 (*N* = 14). There was an increase in frequency count of participants who gave the highest ranking of six for feasibility to either Mindfulness Training or Setting Realistic Goals from T0 (*N* = 13) to T2 (*N* = 15).

**Table 5:** Total ranked scores (range of scores from 16 to 96) for Usefulness and Feasibility at baseline (T0), after round robin one (T1) and round robin two (T2) discussions (*N* = 16)

	USEFULNESS			FEASIBILITY		
	T0	T1	T2	T0	T1	T2
Mindfulness Training	74	81	90	64	75	68
Setting Realistic Goals	79	79	77	73	80	76
Mobile Phone Apps	36	29	29	60	51	65
Motivational Interviewing	50	40	45	51	42	39
Smoke Free Environments	56	61	52	56	60	51
Support Groups	40	46	44	34	31	32

Mobile phone apps were clearly ranked the least useful intervention and Support Groups were ranked the least feasible strategy at T0 and T2. The number of participants that assigned the lowest usefulness ranking of one for mobile phone apps increased from (*N* = 6) at baseline to (*N* = 9) T2. Approximately half the participants assigned the lowest feasibility ranking of one at T2 for Support Groups (*N* = 7). Smoke Free Environments and Motivational Interviewing were the two middle-range interventions throughout the workshop. Perceived feasibility of Motivational Interviewing declined progressively throughout the workshop, while rankings of its usefulness decreased at T2 compared to T1. Smoke Free Environments, for both feasibility and usefulness, experienced an increase from T0 to T1 and then a decrease from T1 to T2.

### Qualitative data

The following themes highlighted the contextual influences of smoking, and the participants’ perceptions of the acceptability of each of the six interventions.

#### Smoking in context

A few participants talked explicitly about how the wider social contexts of their lives, such as a family crisis, pulled them back into smoking despite attempts at cessation. This lens influenced participant perspectives around whether interventions were feasible or useful.

Female smoker: *“I didn’t smoke for the whole of June, but I had a family crisis that made me start smoking again.”*

Male smoker: “*… either I stumbled into a smoking girlfriend, or there was a party and I had just one cigarette. And, recently I stopped for about a week, and then got hit by this flu, so I couldn’t exercise. So, then I’ve been smoking again for 6 days. It’s life.”*

Participants also talked about relationships in their lives where a harsh socio-cultural milieu around smoking led them to develop self-protective behaviours around their smoking cessation journey. Three smokers, for example, describe being ‘shot down’ by friends and professionals for cutting down their level of smoking to amounts they felt good about:

Male smoker: *“For me, it has to be an internalised thing. Because, non-smokers judgy. Ex-smokers judgy. And, that’s the last thing you want when you’re giving up smoking, because even though, if you cut down the amount you’re smoking by 2 cigarettes a day, you achieve that for a week, you feel good, you feel good about yourself. And then you mentioned it to someone else and they just shoot down your face ‘but you’re still smoking’!”*

Female smoker: *“I mean, I’d be pleased with myself. Because I’ve cut down, down to 15, 20 on a bad day; when I was up to you know 40 or something. And I was pleased with that. But the doctors don’t give you any credit anyway, you know, all the people that are supposed to support you, or the cardio or the dietician or whatever, it’s a bit of a mongrel actually.”*

Female smoker: *“See, I suffer from pain all the time, and have for 20 years and it’s getting worse as I’m getting older. And, the cigarettes I miss every second, well, not every second, but I miss it. So, the only reason that I’ve really given it up is 1) health and 2) that I can’t afford it. But I do have the occasional cigarette with my friends, who smoke. And, you know, that, I classify myself as giving up, because I’m not smoking 30 a day. But my boys tell me that’s not giving up because I’m still having the occasional cigarette.”*

The harsh social environment in which people were attempting to quit smoking provided a backdrop to the participants’ evaluation of the six interventions. Throughout the discussion, participants articulated the need to select cessation interventions that were feasible and useful in their own situation and one participant commented that this may not necessarily only include cessation strategies that were outlined in the NGT workshop. One female smoker outlined that self-kindness and self-forgiveness were critical features of any cessation journey regarding being a battler in dire financial and social circumstances, which was not represented explicitly in any of the six interventions:

“*… every cigarette that they sort of don’t have, to sort of celebrate that. And the other thing is, never stop giving up. So, if you’re sort of wanting to give up, but then if you start it again, don’t pick on yourself, just keep, keep that going. Every time.”*

There was explicit consensus that the selection and use of only one intervention would be an insufficient response given the intensity of the gravity with which they were pulled back towards smoking. Both ex-smokers were a couple who had quit 4 months prior to the workshop, and they emphasised the need to use multiple interventions concurrently to achieve their cessation. They positioned effective cessation strategies as interlinked and important drivers to their ability to successfully quit. A desire to intertwine several interventions was asserted by others in the group like one female smoker, who suggested an affordable ‘wellness clinic’ could help smokers to quit:

*“I’d love a wellness clinic for a week, with all those supports in the one place. But, some of that promotional anti-smoking money, go towards something more permanent and effective, and then people can attend the clinic at low cost or minimal cost; similar to heroin addict or that sort of thing. So, to have it all there.”*

#### Mindfulness training, setting realistic goals, and their combined use

There was agreement from writing in silence to the end of the second group discussion that Mindfulness Training and Setting Realistic Goals were perceived as the most useful and feasible cessation interventions, even from those participants who had not used these interventions before. There were varied reasons as to why participants were in favour of these two interventions, for example a female smoker reported at baseline that Mindfulness Training offered a “*conscious understanding and control … that repositioned control to the self*”. Two other female smokers reported respectively at baseline that Setting Realistic Goals was the “most *sensible approach”,* and “*your own personal gain feels great once achieved, small to begin with but end result feels great*”.

Some reported that these two interventions should be used in combination and/or gave reasons why they should be included in RISC. At baseline, Mindfulness Training was the only intervention to have positive statements made by every participant; and all but two participants had positive statements about Setting Realistic Goals. For example,

Female smoker*: “I have never heard of this type of ‘treatment’ (referring to Mindfulness Training). I feel that this might be done in conjunction with setting a realistic goal.”*

Female smoker*: “Setting goals I think could benefit in quitting and staying off smokes goals and keep going in mindfulness.”*

At the conclusion of the workshop (T2), one male smoker suggested that Mindfulness Training and Setting Realistic Goals were both ranked and rated as the most useful interventions by many participants because they directly encourage strategies that are related to an individual’s inherent abilities:

“*Very intrinsic … to do with the self-ability of the person as to whether they can achieve that, and whether they can keep going with that.*”

At the conclusion of the workshop, some participants identified which smoking cessation interventions they wanted to learn more about, or use – one female smoker articulated that she felt using two interventions together will benefit her the most:

“*I’m quite interested that a lot of, well, mindfulness wasn’t as well-known as all the other terms we have there, yet within discussions it’s come through as most useful … I’m already quite clear, from this session today, on what I need to do to be able to meet the mindfulness, and realistic goals. Which I’m very happy about, and I didn’t expect that …*”.

#### Support groups

Several participants identified that Support Groups were not as favourable to include in an intervention for them because they were time-consuming or didn’t fit with personal preferences. Other participants reported concerns around social anxiety/phobia from participating in groups; although, one female smoker suggested at baseline that this may be ameliorated by using an online support group format rather than face-to-face:

“*… I don’t like large groups. My life is very busy so finding time is very hard.”*

Another female smoker at group discussion two*:* “*… to have a support group you’re locked into to a time and place, sometimes. If it’s online, I mean I know, there’s a lot of places that have online, and you can hook in, or something. That might be less intimidating the actually physically, the actual physical presence rather than, on the internet”.*

While a few participants believed Support Groups would offer a positive outcome (male participant at baseline: *“Great support for those who need the guidance, examples and experiences of others”),* Support Groups that consisted of current smokers were reported as the least feasible and not very useful throughout the workshop by several participants because they were perceived as smoking environments that would provide constant reminders and cues about smoking. The following quote describes hesitation about Support Groups based on perceived expectations rather than past experiences.

Female smoker*: “I don’t believe that putting a group of addicts in the room at the same time, if that makes any sense, because I think people can feed off each other.”*

#### Mobile phone apps

Throughout the workshop there was strong consensus that the mobile phone apps were not useful. This assessment was often based on previous experiences. Much of the discussion centred on first generation unidirectional smoking cessation mobile phone apps that focussed on only receiving text messages, rather than the more recently developed bi-directional mobile phone apps that are focussed on tailoring socially interactive messages. This may have influenced participant perceptions of their usefulness. Other concerns included the cost of owning a mobile phone and securing Wi-Fi connection, the difficulties associated with mobile phone apps disrupting phone functioning, and that smoking cessation text messages could serve as an unhelpful cue to smoke:

“***Ok, are you surprised about the mobile app one (during round robin two the facilitator refers to lowest usefulness ranking from first round robin)?”***

Chorus: *“No”! < everyone laughs>.*

Male smoker: *“They breakdown, they ruin a whole bunch of your … there’s no games in mobile apps!”****.***

Female smoker: *“I didn’t like the fact that it sent me messages to remind me about cigarettes, … , because that just reminded me I wasn’t smoking.”*

Ex-smoker: *“And wouldn’t you be subject to like a Wi-Fi data connection or something like that, and what if you’re not in an economic position where you can afford Wi-Fi, or you don’t have, you don’t have phone credit. So, you can’t check in, and you can’t text back. That cuts out a massive group of people that for them, it’s just they can’t access it. “.*

#### Motivational interviewing

Motivational Interviewing was identified by participants as a smoking cessation intervention they were unfamiliar with. One participant articulated that they had never seen this type of service advertised, and another participant suggested that this intervention had been ranked lowly because of limited prior exposure. Table 3 shows only one participant had used Motivational Interviewing.

Female smoker: *“I mean I’ve actually never seen a smoking counsellor ever advertised anywhere. I’ve seen drug counsellors. I’ve seen other, like you know, like domestic counsellors or whatever else. But I’ve never seen someone that actually focuses on, on smoking.”*

Female smoker: “*I’m surprised about the motivational. As low as 2*.*”*

Another female smoker in response to the quote immediately above: *“Can I just say … not many people would have had experienced that type of value, of that, and how it actually works? …*”.

Others reported Motivational Interviewing as being potentially difficult to successfully implement because the counsellor would need to be able to make a ‘personal connection’ with smokers, and that they would have to be seen as trustworthy.

Male smoker at the second group discussion: *“Because it’s a two-way thing as well, you’ve got to be able to trust that person, and they’re supposed to be motivating you, then you’ve got to be able to hold them in some sort of a value as well.”*

A further influence on the rankings around Motivational Interviewing might have been the negative experiences that most (if not all) participants had with health professionals or counsellors around smoking cessation. Many participants identified that Quitline staff (an Australia wide smoking cessation telephone counselling service) lacked empathy, kindness and understanding of the challenges involved in quitting, and also the ability to help them practically with their goal of giving up smoking. One female smoker talked about how she was made to feel terrible by a Quitline worker because of her struggle to give up:

*“I’ve got an experience with the Quitline, I rang the Quitline because I really wanted to give up, and then I wasn’t able to give up, and she got really cross with me, and it made me feel really terrible. So there’s that, about the Quitline, I just didn’t feel like I was supported at all … she was pretty derogatory with me and I, just felt well, you know, she just doesn’t understand how hard it is.”*

In response to this, another female smoker suggested that Quitline workers should be ex-smokers, because it would help them understand what it was like to be giving up:

*“I believe that the Quitline should all be ex-smokers so they have the empathy for smokers, because the lady I was talking to had never smoked. And I thought, what are you doing this job for if you’re not on our level. And she, I couldn’t believe when, I really think that they, I really feel they need to be, sorry, mandatory for them to counsel other people, to have been through it themselves.”*

In addition to the potential difficulties of engaging other, potential unknown people in a cessation attempt amidst a harsh cultural milieu that condemned smoking, another female smoker identified that cessation interventions that depended less on professional support were most useful because you don’t have to go elsewhere to seek them:

*“They are long term tools. You don’t have to go elsewhere to seek them, they’re within you, and, you already, if you’ve been through, and you have the techniques.”*

#### Smoke-free environments

Smoke-free environments were viewed by participants as something that was for the most part outside of their control, and therefore was insufficient on its own to include in an intervention for cessation. One female smoker for example talked about transitioning into study which had provided her with a smoke-free environment that she hoped would help her in ceasing to quit. However, she also identified that she was needing to draw on other strategies when a smoke free environment wasn’t possible:

*“Yeah, because you can’t guarantee you won’t be around people that smoke. Like, my whole family smokes, and I’m kind of, getting, preparing myself for when I go see my family, because it’s like, it’s a fortnightly thing, and it’s like, you can’t just think ‘I can’t just be around smokers anymore’.”*

One female smoker talked about a recent experience where she was able to go nine days without a cigarette in a medical service while using patches in a smoke free environment, but when she re-entered her customary life, she wasn’t able to maintain this cessation attempt:

*“I just had major surgery, and I had patches, like recently, … Yeah, I went 9 days without a cigarette. Yeah, fantastic. Walked straight out and went ‘I want a smoke’, because I saw someone smoking.”*

One male smoker talked about needing smoke-free environments to be introduced at the right time in order to prevent relapse; so, while this acknowledges they have some utility they are always being employed in conjunction with other quit strategies:

*“The day, like when I’m building up to quit, it’s not really that important. But once I’ve got a few days without tobacco in my system, then it’s crucial to stop me from having a relapse, you know. Like, that’s when I try to avoid pubs and, you know gatherings where people smoke etc. So, depends on when.”*

## Discussion

Ranking and rating highlighted that the group approached consensus on Mindfulness Training and Setting Realistic Goals as the two most feasible and useful interventions. For both the final total rankings and the Likert rating measures there was often a clear margin between these two RISC components’ scores for both feasibility and usefulness and the other proposed interventions. These two RISC components were also ranked highest by a high percentage of participants. There was a large increase in scores from baseline to post round robin two regarding how useful Mindfulness Training was perceived to be, which was demonstrated by the statistically significant increase in the Likert score as well as the increase in total ranking score. These are important findings because there is a small amount of recent research that has found other interventions, such as mobile phones that employ text messages and proactive counselling [46] or mindfulness training [47] for smokers, might be effective for lower SES groups of smokers in the short-term but they have not listened to the voices of this target population regarding how they perceive the usefulness and feasibility of such strategies, which is likely to impact on their effectiveness in the longer-term.

The qualitative data in this study demonstrated a variety of reasons why participants have found it difficult to maintain smoking abstinence once they had quit. These reasons are related to the wider and complex social context of their lives where there are many pervading triggers and cues that have successfully pulled participants back to smoking. This highlights the need for a contextualised and tailored smoking cessation intervention. The qualitative data provided an understanding about why the decisions about the preferred RISC components were made before, during, and post the workshop deliberations. There were reasons given why Mindfulness Training was preferred (for example, that it would offer a sense of control), and there were separate reasons why Setting Realistic Goals was preferred (for example, that it would assist in helping achieve personal gains). The reason why the ranking and Likert scores of usefulness and feasibility mostly increased throughout the workshop maybe explained in one of the female smoker’s comments. She was less aware of what the concept of mindfulness training represented before the workshop but found the positive discussions in round robin 1 and 2 on mindfulness had changed her opinion and scores to favour mindfulness as “*most useful”* by the end of the workshop, which has resulted as part of the deliberative democratic process inherent in the NGT. However, some participants specifically reported that Mindfulness Training and Setting Realistic Goals be combined because they were inextricably ‘linked’ and could complement each other. There was clear consensus that regardless of the type of cessation intervention employed, more than one cessation intervention should be used and these interventions should be interconnected. Given authors such as Michie et al. [23] advocate for fewer techniques to be employed in smoking cessation interventions for smokers from lower socioeconomic populations; and that Mindfulness Training and Setting Realistic Goals were clearly perceived as the most feasible and useful as well as linked, one might consider offering just these two interventions [23].

The qualitative data offered a variety of insights into the impact of context on smoking behaviour. For example, cessation strategies that relied heavily on support from others were seen as more problematic because a trusting relationship would need to be established and that they are likely to be less sustainable. For some participants, their experience of mistrust was so extreme that they felt that others were working against them quitting smoking, that they had been ‘shot down’. One participant stated that Motivational Training and Setting Realistic Goals were perceived as the most useful and feasible by many at the end of the workshop because these interventions were ‘intrinsic’ and are aimed at building internal properties regarding “*self-ability*” or self-determination; they enhanced internal resilience. Existing research confirms the importance of self-compassion for quitting with Kelly et al. [48] suggesting that a self-compassionate stance could enhance smoking self-regulation. A recent meta-analysis has confirmed this relationship for health promoting behaviour in 15 different samples [49]. Our study participants confirmed that being kind to oneself, in spite of societal attitudes to smoking, was a key component of being able to persist with cessation, and interventions which amplified self-reliance in a self-compassionate way were most acceptable to participants and should be considered as critical to any intervention for cessation.

Our research contributes to this field of research in two major ways. First, it was novel in using theoretical knowledge drawn from the psychosocial interactive resilience model to influence conception, development and design of a smoking cessation intervention for individuals from lower SES populations. Second, it generated feedback from individuals within the target population on conceivable components for a smoking cessation program suggested beneficial in the current literature.

### Limitations

As is the case with most NGT studies, the small sample size prevents generalisability of the findings to the wider population and limits the statistical power of quantitative analyses. Indeed, it is not always clear when consensus has been reached. However, it is possible to argue that agreement has been approached when considering both qualitative data and the descriptive statistical analyses. As is the case with many recruitment approaches there was the potential that not all vulnerable groups of smokers were included because our intention was to capture a broad-based lower SES population, rather than target specific groups. There was intended to be a balanced mix of both contemplative current smokers and recent ex-smokers but the majority of participants were current smokers. Therefore, it was not possible to directly compare the perceptions of the two sub-groups. Further study involving larger representations in each group, including former smokers with longer durations of smoking cessation, could build our understanding of smoking cessation interventions most likely to be effective in populations living in lower socioeconomic circumstances.

## Conclusion

The NGT is a useful method to help develop a health intervention as it allows people from target populations to voice their opinions on what the components should be and how best to implement the program. Some of the ways in which mixed methods have been used in the past have been criticised [50]; but, in the context of using the NGT, it can prove valuable whereby the qualitative data may complement and extend our understanding of the quantitative data. This is because its purpose is not always limited by small sample sizes and such approaches can assist in developing a greater understanding of how consensus was approached [51]. The quantitative results of this study presented Mindfulness Training and Setting Realistic Groups as both useful and feasible strategies to be employed in RISC. The qualitative analyses largely supported the quantitative findings and also raised important questions, identified barriers, and (in some cases) offered solutions by and for smokers from lower SES populations who are contemplating quitting. The results of this study provide valuable knowledge regarding tailoring RISC for lower SES populations. This knowledge is likely to be important in helping develop a RISC that can be trialled with a large representative sample. We anticipate that this research, which aims to enhance resilience via mindfulness training and setting realistic goals, could have implications for further research in other health related addictive behaviours and other health promotion interventions, such as alcoholism. That is, RISC could be adapted as an intervention to remove other addictions that can harm the health and wellbeing of lower SES populations.

## Supplementary information

**Additional file 1.** Figure S4 Background information sheet

**Additional file 2.** Figure S5 Ranking resilience strategies on usefulness and feasibility

**Additional file 3.** Figure S6 Nine-point Likert rating scales of how useful and feasible each of the strategies were perceived

## Data Availability

The data analysed during this study are available from the corresponding author on reasonable request.

## Abbreviations

AUD: Australian Dollars
NGT: Nominal Group Technique
RISC: Resilience Intervention for Smoking Cessation
SEIFA: Socio-Economic Index for Areas
SES: Socioeconomic status
