# Supplementary material for: Using a nominal group technique to approach consensus on a resilience intervention for smoking cessation in a lower socioeconomic population
Source: BMC Public Health. 2019 Nov 27;19:1577. doi: 10.1186/s12889-019-7939-y (PMC6882049; doi:10.1186/s12889-019-7939-y)
Supplement: Supplementary file 1 — Additional file 1. Figure S4 Background information sheet [file 12889_2019_7939_MOESM1_ESM.docx]

**Planning your ideal quit smoking program**

Thanks for coming today. We want to understand more about what might be useful for people wanting to quit smoking. Please fill in details below, so we can understand your smoking history. This will help us to better understand your preferences for an ideal quit smoking program.

First name: ___________________________________________

Age: _________ Gender: Male / Female (please circle)

When did you start smoking?: _______________________________________

How old were you when you started smoking? ____________________

How many cigarettes per day on average did you smoke?: _________________

Have you tried any of these strategies before? (circle Y or N)

Motivational interviewing Y / N

Support groups Y / N

Mindfulness training Y / N

Setting realistic goals Y / N

Improve access to smoke-free environments Y / N

Mobile phone apps Y / N

Nicotine replacement (lozenges, patches, etc) Y / N

If you’ve been able to quit before, can you tell us how, when, and for how long?:

______________________________________________________________

______________________________________________________________02

If you’re still smoking, can you tell us your reason for wanting to quit now?:

______________________________________________________________

______________________________________________________________

Thank you! Please hand back to one of the researchers

Figure 4: Background information sheet
