# Supplementary material for: Using a nominal group technique to approach consensus on a resilience intervention for smoking cessation in a lower socioeconomic population
Source: BMC Public Health. 2019 Nov 27;19:1577. doi: 10.1186/s12889-019-7939-y (PMC6882049; doi:10.1186/s12889-019-7939-y)
Supplement: Supplementary file 2 — Additional file 2. Figure S5 Ranking resilience strategies on usefulness and feasibility [file 12889_2019_7939_MOESM2_ESM.docx]

Please **rank** the strategies in terms of their usefulness to you. The most useful will be given a 6, the next most useful a 5, and so on.

6. (most useful) ______________________________________

5. ______________________________________

4. ______________________________________

3. ______________________________________

2. ______________________________________

1. (least useful) ______________________________________

Please **rank** the strategies according to what would be easy to put into practice and what would be the most difficult. The easiest will be given a 6, the next easiest a 5, and so on.

6. (easiest) ______________________________________

5. ______________________________________

4. ______________________________________

3. ______________________________________

2. ______________________________________

1. (most difficult) ______________________________________

Figure 5: Ranking resilience strategies on usefulness and feasibility
