# Supplementary material for: Using a nominal group technique to approach consensus on a resilience intervention for smoking cessation in a lower socioeconomic population
Source: BMC Public Health. 2019 Nov 27;19:1577. doi: 10.1186/s12889-019-7939-y (PMC6882049; doi:10.1186/s12889-019-7939-y)
Supplement: Supplementary file 3 — Additional file 3. Figure S6 Nine-point Likert rating scales of how useful and feasible each of the strategies were perceived [file 12889_2019_7939_MOESM3_ESM.docx]

First name: ___________________________________________

Please rate how **useful** you think each potential component/ strategy would be for helping you to quit smoking.

Please circle a number on a scale from **1-9** on how **useful** you think each strategy discussed today would be to help you quit smoking (a low number represents not useful and a high number represents very useful).

**Mindfulness Training**

How **useful** would this strategy be in helping you quit smoking.

Not useful Neutral Very useful

1 2 3 4 5 6 7 8 9

**Motivational Interviewing**

How **useful** would this strategy be in helping you quit smoking.

Not useful Neutral Very useful

1 2 3 4 5 6 7 8 9

**Setting Realistic Goals**

How **useful** would this strategy be in helping you quit smoking.

Not useful Neutral Very useful

1 2 3 4 5 6 7 8 9

Please circle a number on a scale from **1-9** on how **useful** you think each strategy discussed today would be to help you quit smoking (a low number represents not useful and a high number represents very useful).

**Improve Access to Smoke-Free Environments**

How **useful** would this strategy be in helping you quit smoking.

Not useful Neutral Very useful

1 2 3 4 5 6 7 8 9

**Mobile Phone Apps**

How **useful** would this strategy be in helping you quit smoking.

Not useful Neutral Very useful

1 2 3 4 5 6 7 8 9

**Support Groups for Smokers to Quit**

How **useful** would this strategy be in helping you quit smoking.

Not useful Neutral Very useful

1 2 3 4 5 6 7 8 9

Please rate how **difficult or easy it would be to put into action or practice** each potential strategy for helping you to quit smoking.

Please circle a number on a scale from **1-9** on how **difficult or easy it would be to put into action or practice** each strategy discussed today in relation to helping you quit smoking (a low number represents difficult and a high number represents easy).

**Mindfulness Training**

How **difficult or easy** would this strategy be to put into action or practice to help you quit smoking?

Difficult Neutral Easy

1 2 3 4 5 6 7 8 9

**Motivational Interviewing**

How **difficult or easy** would this strategy be to put into action or practice to help you quit smoking?

Difficult Neutral Easy

1 2 3 4 5 6 7 8 9

**Setting Realistic Goals**

How **difficult or easy** would this strategy be to put into action or practice to help you quit smoking?

Difficult Neutral Easy

1 2 3 4 5 6 7 8 9

Please circle a number on a scale from **1-9** on how **difficult or easy it would be to put into action or practice** each strategy discussed today in relation to helping you quit smoking (a low number represents difficult and a high number represents easy).

**Improve Access to Smoke-Free Environments**

How **difficult or easy** would this strategy be to put into action or practice to help you quit smoking?

Difficult Neutral Easy

1 2 3 4 5 6 7 8 9

**Mobile Phone Apps**

How **difficult or easy** would this strategy be to put into action or practice to help you quit smoking?

Difficult Neutral Easy

1 2 3 4 5 6 7 8 9

**Support Groups for Smokers to Quit**

How **difficult or easy** would this strategy be to put into action or practice to help you quit smoking?

Difficult Neutral Easy

1 2 3 4 5 6 7 8 9

Figure 6: Nine-point Likert rating scales of how useful and feasible each of the strategies were perceived
